# Idiopathic Juvenile Osteoporosis: A Case Report and Literature Review

**DOI:** 10.7759/cureus.68361

**Published:** 2024-09-01

**Authors:** Shunsuke Shimazaki, Junichi Sato

**Affiliations:** 1 Department of Paediatrics, Funabashi Municipal Medical Center, Funabashi, JPN

**Keywords:** rare bone disease, recurrent fracture, idiopathic juvenile osteoporosis, bone mineral density, bisphosphonate

## Abstract

This case report describes the rare occurrence of idiopathic juvenile osteoporosis (IJO) in an 11-year-old boy with bone fragility and fractures, particularly in the thoracic and lumbar vertebrae. After excluding discernible underlying causes, the diagnosis was confirmed using clinical and radiological assessments. Treatment commenced with oral bisphosphonates, leading to notable bone mineral density (BMD) improvements and the absence of subsequent fractures. IJO presents diagnostic challenges owing to its multifaceted nature, necessitating the exclusion of other common causes of pediatric osteoporosis. Although the pathophysiology of IJO remains poorly understood, this case underscores the potential efficacy of bisphosphonate therapy in managing the condition and improving patient outcomes. Notably, the patient's symptoms ameliorated as puberty commenced, aligning with the typical IJO patterns reported in the literature. Although the long-term impact of bisphosphonate treatment in pediatric IJO cases warrants further investigation, this case exemplifies the potential to enhance the quality of life of affected individuals.

## Introduction

Osteoporosis is frequently prevalent in adults but is not common in children. Most cases of osteoporosis in children occur secondary to glucocorticoid therapy, chronic inflammation, or primary genetic conditions involving osteogenesis imperfecta (OI), Ehlers-Danlos syndrome, and Marfan syndrome [1]. Idiopathic juvenile osteoporosis (IJO) is a rare disease that develops before puberty. It usually resolves in puberty, but bisphosphonate therapy improves biochemical and radiological findings [2-4]. Herein, we describe the management and six-year follow-up of a boy with IJO.

## Case presentation

An 11-year-old boy presented with a back pain. He had no significant medical history of recurrent fractures or primary illnesses. No associations with trauma were observed. The patient had no family history of recurrent fractures or osteoporosis. Physical examination revealed a height of 131.6 cm (-1.54 standard deviation (SD), 7th percentile), a weight of 34.4 kg (-0.12 SD, 42nd percentile), an arm span of 131 cm, and a bilateral testicular volume of 3 mL on both sides. He had no blue sclerae, hyperelastic skin, or alternations of tooth enamel. He was prepubertal. Plain radiographs of the spine revealed multiple thoracic and lumbar vertebral fractures (Th10, L1, and L2). He had no history of injuries that could cause fractures.

**Figure 1:**
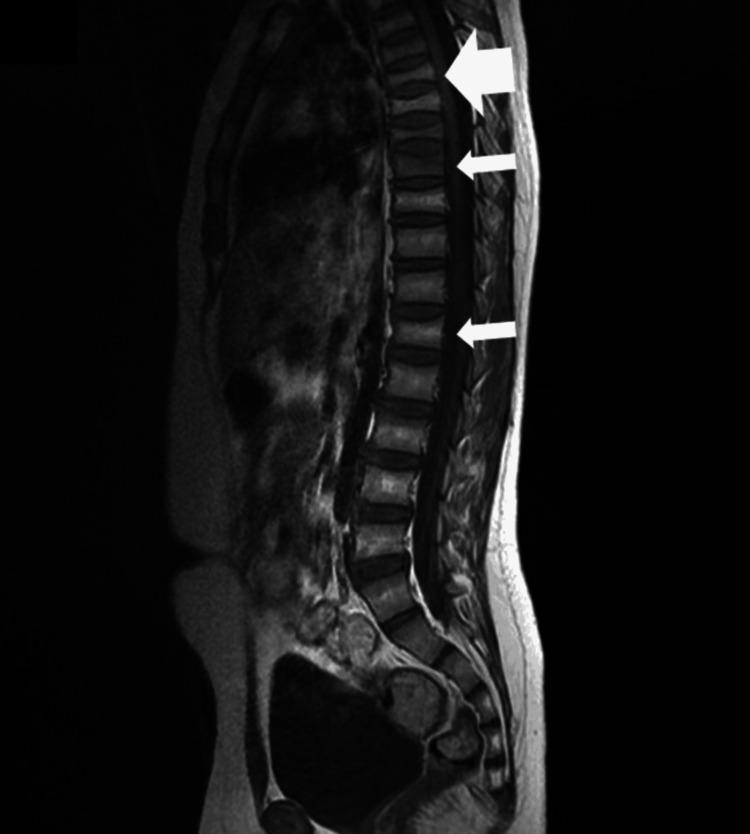
A sagittal T1 contrast-enhanced magnetic resonance imaging demonstrating multiple previous vertebral compression fractures.

Magnetic resonance imaging of the spine was performed to evaluate the whole spine and abnormalities, such as tumors and metastases. However, we did not find them without vertebral compression fractures (Figure 1). The thickness of his long bones was normal.

Complete blood cell counts and electrolyte levels were normal (Table 1). The patient’s laboratory test results showed normal levels across the following parameters: serum calcium at 9.3 mg/dL, phosphorus at 4.7 mg/dL, bone alkaline phosphatase at 185 IU/L (using the International Federation of Clinical Chemistry and Laboratory Medicine method), and intact parathyroid hormone at 17 pg/mL. The serum 25-hydroxy vitamin D level was also within the normal range. The urine N-telopeptide of type Ⅰ collagen (NTx) level was significantly elevated at 570.5 nmolBCE/mmol・Cre (normal range: 13.0-66.2 nmolBCE/mmol・Cre), indicating heightened bone resorption. Whole-body densitometry (using dual-energy X-ray absorptiometry, DXA) revealed a bone mineral density (BMD) of 0.293 g/cm^2^ (Z-score -7.2) at the lumbar spine and 0.404 g/cm^2^ (Z-score -5.8) at the left proximal femur.

**Table 1:** Serum and urinalysis laboratory data of the patient before treatment WBC: white blood cells; RBC: red blood cells; Ca: calcium; P: phosphorus; ALP: alkaline phosphatase; BUN: blood urea nitrogen; Cr: creatinine; CRP: C-reactive protein; 25(OH)D: 25-hydroxyvitamin D; PTH: parathyroid hormone; TSH: thyroid-stimulating hormone; FT3: free triiodothyronine; FT4: free thyroxine; ACTH: adrenocorticotropic hormone; NTx: N-telopeptide of type I collagen

Index	Results	Reference range
WBC (/μL)	5500	4500-13500
RBC (/μL)	5.30×10^6^	4.27-5.70×10^6^
Hemoglobin (g/dL)	13.5	13.5-17.6
Ca (mg/dL)	9.3	8.7-10.0
P (mg/dL)	4.7	3.8-5.5
ALP (IU/mL) (IFCC method)	185	165-525
BUN (mg/dL)	10.6	6.0-20.0
Cr (mg/dL)	0.35	0.35-0.58
CRP (mg/dL)	0.04	0-0.2
25(OH)D (ng/mL)	21	20-100
intact PTH (pg/mL)	17	10-65
TSH (µU/mL)	1.283	0.62-3.36
FT3 (pg/mL)	3.11	2.78-4.90
FT4 (ng/dL)	1.04	1.02-1.52
ACTH (pg/mL)	18.1	10.5-38.1
Urine NTx (nmolBCE/mmol・Cre)	570.5	13.0-66.2

The absence of a discernible underlying etiology of IJO was confirmed based on these observations. The patient was orally administered alendronate (35 mg/week). After the commencement of treatment, there was no incidence of fragility fractures. The patient manifested puberty at 12 years of age. His BMD normalized at 14 years of age (three years after treatment initiation; Table 2). No fractures were detected after treatment. The treatment was completed in approximately four years. His bilateral testicular volume had increased to 14 mL. Over a year has elapsed since the conclusion of treatment, and no fractures have been detected. He avoids excessive physical activity but maintains a normal school life.

**Table 2:** BMD and Z score for lumbar spine and left proximal femur BMD: bone mineral density

Time since treatment (month)	Lumbar spine		Left proximal femur	
	BMD (g/cm^2^)	Z score	BMD (g/cm^2^)	Z score
0	0.293	-7.2	0.404	-5.8
5	0.396	-5.8	0.369	-6.8
11	0.464	-2.8	0.450	-5.3
17	0.590	-3.5	0.568	-3.5
22	0.666	-2.6	0.628	-2.8
27	0.749	-1.8	0.662	-2.5
33	0.797	-1.6	0.714	-2.2
39	0.846	-1.4	0.762	-1.9

## Discussion

Diagnosis of IJO

Osteoporosis in children and adolescents can be categorized as primary or secondary. Patients with primary osteoporosis have severe bone fragility that causes inherent skeletal defects with abnormal bone tissue composition. They are heterogeneous groups with various skeletal and extraskeletal characteristics [5]. Management of osteoporosis is important because it may lead to severe bone diseases and conditions. OI is well-known as a genetic disease associated with primary osteoporosis. It is a group of clinically diverse disorders that primarily affect the synthesis of adequate amounts of type I collagen [6]. However, no physical findings specific to OI, such as blue sclera, were observed in our patient. Patients with secondary osteoporosis have underlying systemic conditions and are on specific medications. Common causes include glucocorticoid treatment, systemic inflammation, deficiency in sex steroids, and hematopoietic skeletal effects [7].

IJO is a rare disease that occurs between eight and 14 years of age, and its symptoms may improve during puberty [8,9]. The pathophysiology of the IJO remains poorly understood and it poses diagnostic challenges due to its complexity. Initially, it is imperative to rule out other common primary or secondary forms of osteoporosis. Subsequently, as in our patient, a diagnosis of IJO was made based on radiographic and clinical findings.

BMD to assess osteoporosis

BMD is one of the important indicators used in the diagnosis of osteoporosis [5]. It is a measurement that indicates the amount of minerals, primarily calcium, in a defined area of bone. It reflects the strength and density of the bones and is used as a key indicator in assessing the risk of fractures. It can be measured using various techniques, with DXA being the most common method. The BMD of our patient was monitored using DXA.

Pathophysiology of IJO

Children with IJO experience symptoms, such as long bone fractures, back pain, and difficulty walking [10]. These symptoms usually begin with puberty. IJO involves a two-fold dysfunction in cancellous bone formation [11]. First, fewer remodeling cycles were initiated. This will not resolve bone integrity in the short run because the recruitment of osteoblast and osteoclast teams is similarly diminished. Second, the amount of bone formed in each remodeling cycle is noticeably diminished. These outcomes involved a reduction in the thickness of the mature trabeculae and a potential decrease in the formation of secondary trabeculae in the metaphysis. Bacchett et al. reported a cross-sectional analysis using quantitative CT scans and bone histomorphometry that revealed decreased bone turnover and trabecular and cortical Z-scores for bone density [9]. Mutations in LRP5 are found in pediatric patients with primary osteoporosis [12-14]. Stürznickel et al. reported that LRP5 and LRP6 variants may contribute to severe early onset osteoporosis (EOOP) in a study of 372 EOOP patients [15]. It is anticipated that there will be an accumulation of knowledge through genetic research in the future. Currently, the diagnosis of IJO relies on clinical assessment and the exclusion of alternative causes of osteoporosis.

Management and treatment of IJO

Most patients with IJO experience resolution of their symptoms; however, a few experience recurrent fractures, which may reveal severe kyphosis and skeletal deformities [16,17]. Limited information is available regarding the effect of bisphosphonates on pediatric and adolescent patients with IJO. In children with cerebral palsy, bisphosphonates significantly improve BMD [18]. Using bisphosphonates has the advantage of reducing pain, decreasing fracture recurrence, and lowering the risk of future disability due to permanent deformities [17]. Bisphosphonates can be administered either orally or intravenously, with numerous cases reported for intravenous route [14,16,17,19]. However, side effects, such as fever and nasal discharge, may be observed with intravenous bisphosphonate use. Moreover, there is a burden on the child because they must remain still during the medication administration. On the other hand, oral administration can be easily performed at home in children, but esophagitis may sometimes be reported. It is necessary to maintain an upright position for a while after taking the medication. Moreover, oral bisphosphonates exhibit somewhat diminished efficacy, and reports on therapeutic outcomes achieved with oral bisphosphonates remain limited, particularly in cases of milder osteoporosis [20]. However, although our patient’s condition was as severe as that treated with intravenous bisphosphonates, the improvement in BMD achieved with oral bisphosphonate treatment was comparable. The appropriate dosage of oral bisphosphonates in children is unknown. If more data is collected on the use of oral bisphosphonates for IJO, their effectiveness may become clearer.

## Conclusions

We managed a patient with IJO complicated by fragility fractures. By using oral bisphosphonates, we were able to improve his bone density and reduce fractures. IJO is a rare disease, and as a result, the treatment and management strategies remain largely uncertain. It has been suggested that the oral administration of bisphosphonates may be effective. It is important to carefully assess the severity of patients with IJO, and bisphosphonate treatment may be beneficial for such patients. It appears feasible to improve the quality of life of patients with IJO through the ongoing accumulation of insights.

## References

[1] Ward L, Glorieux F (2003). The spectrum of pediatric osteoporosis. Pediatric Bone: Biology and Diseases.

[2] Lorenc RS (2002). Idiopathic juvenile osteoporosis. Calcif Tissue Int.

[3] Krassas GE (2000). Idiopathic juvenile osteoporosis. Ann N Y Acad Sci.

[4] Hoekman K, Papapoulos SE, Peters AC, Bijvoet OL (1985). Characteristics and bisphosphonate treatment of a patient with juvenile osteoporosis. J Clin Endocrinol Metab.

[5] Ciancia S, van Rijn RR, Högler W, Appelman-Dijkstra NM, Boot AM, Sas TC, Renes JS (2022). Osteoporosis in children and adolescents: when to suspect and how to diagnose it. Eur J Pediatr.

[6] Rauch F, Glorieux FH (2004). Osteogenesis imperfecta. Lancet.

[7] Sakka SD (2022). Osteoporosis in children and young adults. Best Pract Res Clin Rheumatol.

[8] Rosen CJ (2000). Pathogenesis of osteoporosis. Baillieres Best Pract Res Clin Endocrinol Metab.

[9] Bacchetta J, Wesseling-Perry K, Gilsanz V, Gales B, Pereira RC, Salusky IB (2013). Idiopathic juvenile osteoporosis: a cross-sectional single-centre experience with bone histomorphometry and quantitative computed tomography. Pediatr Rheumatol Online J.

[10] Smith R (1995). Idiopathic juvenile osteoporosis: experience of twenty-one patients. Br J Rheumatol.

[11] Rauch F, Travers R, Norman ME, Taylor A, Parfitt AM, Glorieux FH (2000). Deficient bone formation in idiopathic juvenile osteoporosis: a histomorphometric study of cancellous iliac bone. J Bone Miner Res.

[12] Hartikka H, Mäkitie O, Männikkö M (2005). Heterozygous mutations in the LDL receptor-related protein 5 (LRP5) gene are associated with primary osteoporosis in children. J Bone Miner Res.

[13] Caetano da Silva C, Ricquebourg M, Orcel P, Fabre S, Funck-Brentano T, Cohen-Solal M, Collet C (2021). More severe phenotype of early-onset osteoporosis associated with recessive form of LRP5 and combination with DKK1 or WNT3A. Mol Genet Genomic Med.

[14] Dabas A, Malhotra R, Kumar R, Khadgawat R (2021). Idiopathic juvenile osteoporosis in a child: a four-year follow-up with review of literature. J Pediatr Endocrinol Metab.

[15] Stürznickel J, Rolvien T, Delsmann A (2021). Clinical phenotype and relevance of LRP5 and LRP6 variants in patients with early-onset osteoporosis (EOOP). J Bone Miner Res.

[16] Melchior R, Zabel B, Spranger J, Schumacher R (2005). Effective parenteral clodronate treatment of a child with severe juvenile idiopathic osteoporosis. Eur J Pediatr.

[17] Baroncelli GI, Vierucci F, Bertelloni S, Erba P, Zampollo E, Giuca MR (2013). Pamidronate treatment stimulates the onset of recovery phase reducing fracture rate and skeletal deformities in patients with idiopathic juvenile osteoporosis: comparison with untreated patients. J Bone Miner Metab.

[18] Kim MJ, Kim SN, Lee IS (2015). Effects of bisphosphonates to treat osteoporosis in children with cerebral palsy: a meta-analysis. J Pediatr Endocrinol Metab.

[19] Tan LO, Lim SY, Vasanwala RF (2017). Primary osteoporosis in children. BMJ Case Rep.

[20] Papakonstantinou O, Sakalidou M, Atsali E, Bizimi V, Mendrinou M, Alexopoulou E (2015). Radiographic and MRI imaging findings of the spine after bisphosphonate treatment, in a child with idiopathic juvenile osteoporosis. Case Rep Radiol.

